# Enhancing unsupervised medical entity linking with multi-instance learning

**DOI:** 10.1186/s12911-021-01654-z

**Published:** 2021-11-16

**Authors:** Cheng Yan, Yuanzhe Zhang, Kang Liu, Jun Zhao, Yafei Shi, Shengping Liu

**Affiliations:** 1 National Laboratory of Pattern Recognition, Institute of Automation, Chinese Academy of Sciences, Beijing, China; 2 School of Artificial Intelligence, University of Chinese Academy of Sciences, Beijing, China; 3 Unisound AI Technology Co., Ltd., Beijing, China

**Keywords:** Medical entity linking, Unsupervised learning, Multiple instance learning

## Abstract

**Background:**

A lot of medical mentions can be extracted from a huge amount of medical texts. In order to make use of these medical mentions, a prerequisite step is to link those medical mentions to a medical domain knowledge base (KB). This linkage of mention to a well-defined, unambiguous KB is a necessary part of the downstream application such as disease diagnosis and prescription of drugs. Such demand becomes more urgent in colloquial and informal situations like online medical consultation, where the medical language is more casual and vaguer. In this article, we propose an unsupervised method to link the Chinese medical symptom mentions to the ICD10 classification in a colloquial background.

**Methods:**

We propose an unsupervised entity linking model using multi-instance learning (MIL). Our approach builds on a basic unsupervised entity linking method (named BEL), which is an embedding similarity-based EL model in this paper, and uses MIL training paradigm to boost the performance of BEL. First, we construct a dataset from an unlabeled large-scale Chinese medical consultation corpus with the help of BEL. Subsequently, we use a variety of encoders to obtain the representations of mention-context and the ICD10 entities. Then the representations are fed into a ranking network to score candidate entities.

**Results:**

We evaluate the proposed model on the test dataset annotated by professional doctors. The evaluation results show that our method achieves 60.34% accuracy, exceeding the fundamental BEL by 1.72%.

**Conclusions:**

We propose an unsupervised entity linking method to the entity linking in the medical domain, using MIL training manner. We annotate a test set for evaluation. The experimental results show that our model behaves better than the fundamental model BEL, and provides an insight for future research.

## Background

In the medical domain, Medical Entity Linking (MEL) is the task of identifying and standardizing mentions in an unstructured medical text, and link the mentions to the unique identities in a given medical knowledge base. There are lots of medical entity types, such as disease, medicine, examination, surgery, symptom, and so on. In our work, we mainly concentrate on the linking of symptom mention in the colloquial Chinese medical consultation context.

For example, there is a patient’s colloquial self-describing Chinese text: 我的孩子昨天拉肚子, 严重恶心、头疼、一直呕吐, 一整晚没睡. 这是食物中毒了么? *(My child had diarrhea last night, with a strong feeling of nausea, head fever, and keep vomiting, did not fall asleep all night. I wonder if it’s food poisoning?)* In this sentence, several symptom mentions can be found such as *diarrhea*, *nausea*, *fever*, *vomiting* and *did not fall asleep*. MEL task is proposed to link those unnormalized symptom mentions to the standard and unambiguous entities in a medical KB.

The widely used (including ontologies, controlled vocabularies) in the medical domain are MeSH [1] (Medical Subject Headings), UMLS [2] (Unified Medical Language System), SNOMED CT [3] (Systematized Nomenclature of Medicine - Clinical Terms), and ICD [4] (International Statistical Classification of Diseases and Related Health Problems). We choose ICD10 as our linking target because it is widely adopted around the world and particularly, it has been used much more often in the Chinese medical context than other KBs. In this example, *diarrhea* should be linked to ICD code *K52.916*, *nausea* to *R11.x02*.

There are two main challenges for MEL. First, most neural entity linking methods heavily rely on the large amount of annotated text which is very hard to construct. The reason is twofold: (a) comparing with entity linking in other domain, medical texts involve patient privacy, identity information and law regulations, which makes it difficult even to obtain plain raw medical texts, especially front-line clinical text data such as medical diagnosis and medical treatment; (b) the annotation of MEL is not an easy job. The annotation task not only requires that the annotator should have professional and comprehensive medical background knowledge to identify the correct mention span, but also requires the annotator to be familiar with various KBs. Second, the linking target of medical entity linking is quite different from traditional KBs such as DBpedia [5], FreeBase [6] and YAGO [7]. Medical KBs are relation sparse and incomplete, which increases the difficulty of MEL, since the various methods of exploitation of target KBs used in the common domain are not feasible [8, 9].

Generally, most entity linking neural network methods [10–12] generally adopt the pipeline framework which involves three-step subprocess. In the pipeline framework, the system first performs named entity recognition (NER), that is, the entity recognition module identifies the mention span of medical concepts of interest in the text. Then the following candidate generation step is responsible for generating a limited-size entity candidate sets selected from a knowledge base or ontology which the mention should be linked to. The last entity disambiguation (ED) step, commonly an entity ranking module estimates the similarity between the candidate entities with context-mention pair, to predict the most appropriate entity for the mention in the context. There may do not exist such an entity in the KB that can align to a given mention, so the entity linking model sometimes needs to predict the possible missing entity, which is called NIL entity [13]. In this paper, we do not deal with the NIL issue, leaving it to future works.

The medical community has published many entity linking datasets in the past decades, covering many categories such as disease, medicine, treatment, gene, protein, microbe, and different domains including clinical text, biomedical science or user generated content. Specifically, in the clinical domain, there are only a handful of published datasets: ShARe2013 [14], SemEval-2014 Task 7 [15], SemEval-2015 Task 14 [16], and the recent MCN dataset [17]. All those clinical EL datasets are constructed by human effort and limited to English. Therefore, unsupervised approaches with little labor cost is needed urgently to utilize the massive raw medical texts like EMRs (electronic medical records), online medical consultation, medical textbooks, and drug instructions.

The recent medical entity linking works heavily relied on supervised methods which evaluates on the public annotated dataset described above, while only a few works explored on the unlabeled datasets. The most recent unsupervised entity linking in medical domain was unMERL [18]. They proposed an unsupervised framework for recognizing and linking medical entities mentioned in Chinese online medical text. Yet, the linking approach in unMERL is a purely statistical approach that considers features like string similarity, entity popularity, and semantic correlation between entities. Le’s work [19] is the closest to our work. They trained the model with the MIL paradigm and introduced a noise detecting classifier trained jointly with the EL model to reduce the impact of noisy data. Their work was designed for the general domain and relied on YAGO to generate the MIL-style training dataset with a distant-supervised approach. They still leveraged the annotated development dataset to train the noise classifier. Yet, a dense English KB like YAGO or a handful of annotated data pairs is inaccessible under many practical application scenarios. Our work needs no annotated data and utilizes the cluster structures of ICD10 to improve the performance.

Facing the above challenges, our motivation is directly constructing a MEL dataset from accessible online Chinese medical consultation with certain unsupervised methods, and build an entity linking model on top of it. Our contributions are as follows:

- We propose a method for constructing a MIL-based entity linking dataset from online colloquial Chinese medical consultation.
- Second, we propose a neural entity linking model by incorporating the cluster information of medical entities in ICD10, which can alleviate the KB sparsity problem.
- Our model achieved 60.34% accuracy, exceeding the fundamental BEL by 1.72%.

## Methods

### Overall architecture

**Fig. 1:**
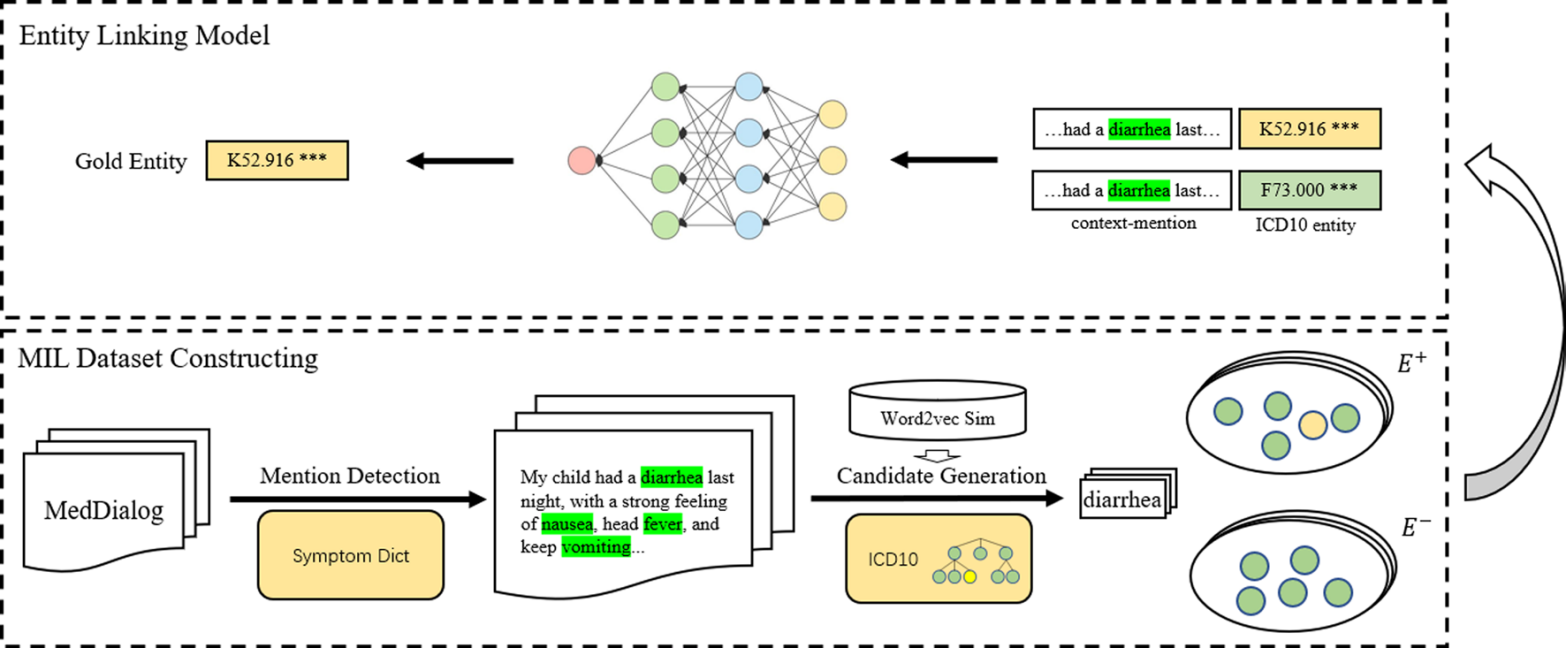
The overall architecture of our approach

Our approach adopts a pipeline architecture as Fig. 1. First, we construct a MIL dataset which consists of mention detection and candidate set generation. Next, a neural network is trained to gap the margin between the positive and negative candidate set, within the MIL frame. We elaborate the components in detail as follows.

### Mention detection

Mention detection is the first step in constructing the training dataset. In our task, we use dictionary-based approaches to find a symptom mention in the raw text constructed from the previous step. The dictionary is crawled from several online Chinese medical wiki websites. The number of concepts crawled is listed in Table 1.

**Table 1:** The number of crawled concepts

Website	Concept num.
FuHe HealthNet [20]	10448
39 HealthNet [21]	7815
99 HealthNet [22]	6060
WYXY.Com [23]	6864
120ask [24]	5740
A+ hospital [25]	6885
Unique total num	12359

The boundary between disease and symptom is not always clearly defined. For example, hypertension can be regarded as a blood disease or a symptom of elevated blood pressure under different circumstances. Thus, along with the data noise, the collected symptom dictionary inevitably contains some diseases. We do not make additional distinctions here and refer to them as symptoms in general terms.

### Candidate generation

Since we frame the MEL task as the multi-instance learning (MIL) problem [26], a MIL-style dataset is needed. In MIL, instead of receiving a set of individually labeled instances, the learner receives a set of labeled bags, each bag containing many instances. A bag is labeled positive if there is at least one instance in the bag is positive and is labeled negative if all the instances in the bag are negative. MIL methods aim at learning classifiers for individual instances.

Once mention detection is finished, the candidate generation module will generate the positive candidate set (*E*^+^, the positive bag) and the negative candidate set (*E*^−^, the negative bag) for MIL. We try to construct such a *E*^+^ that the correct entity should have a high probability to be included, while the entities in *E*^−^ should be as ambiguous as possible with the correct entity.

During the candidate generation process, in order to relieve the learning burden of the linking model, we should try to reduce the size of *E*^+^ under the condition that the gold entity recall is kept at a safe threshold. On the other hand, the negative entity space should be evenly distributed sampled, and it is necessary to select those negative entities that are more similar to the correct entity as much as possible to enhance the ability of the model to distinguish hard entity pairs.

Regarding the generation of *E*^+^ and *E*^−^ we propose two methods:

1. TopK
  For the positive candidate selection, the *TopK* selection method directly selects the *K* entities with the highest similarity between the mention and entity embedding.
  As for the negative candidate selection, there are two approaches. (1) *Random All* randomly selects *K* negative candidates from the remaining ICD10 entities. (2) *Random TopN* first constructs an entity pool of size *N* ($N > K$) with the highest similarity, and then randomly selects *K* negative candidates which are not in the *E*^+^ from the pool. The second negative candidate selection will generate much harder negative samples since the distance between the positive and negative candidate is closer comparing with the first negative selection method.
2. ClusterK
  ICD10 is a tree-like KB with a hierarchical structure that contains the relationship information between entities. For example, “*R50.800 Fever, Other specified fever*” is an ICD10 entity with the code *R50.800*. The first three characters of ICD10, *R50* in this case, are the category (the general type of the injury, disease, or symptom.). The category is followed by a decimal point and the subcategory. The subcategory consisted of up to two sub-classifications (cause, manifestation, location, severity, and type of injury, disease or symptom). Thus, in the example, *80* following the decimal is the first sub-classification following *0* is the second sub-classification.
  In our paper, we define that the entities under the same category and the first sub-classification are treated as an ICD10 *cluster*. We suppose the entities from the same ICD10 cluster should have similar semantics and closer connection which can be used to improve *E*^+^. The *ClusterK* selection method first selects top *N* most similar ICD10 entities as the candidate pool, and then selects entities from the candidate pool according to similarity. Each time an ICD10 entity is selected, the entities in the candidate pool which are in the same cluster are also selected. *K* clusters are selected in this way. The negative candidate selection is the same as *TopK* selection method. Since *ClusterK* selection incorporates the structural information of the ICD10 code, it can reduce the size of the candidate set without significantly reducing recall.

Formally, let context *c* be the entire *l*-word sentence ($w_1,\ldots ,w_l$) from the unlabeled MedDialog [27] dataset and the mention be $m=(w_h,\ldots ,w_k )$, $1 \le h \le k \le l$, which is obtained in the mention detection phase. For a mention *m*, the system will generate the positive candidate set *E*^+^ and negative candidate set *E*^−^. Therefore, we construct a data point $<m,c,E^+,E^->$ from an unlabeled MedDialog sentence which is a tuple of mention *m*, context *c*, positive set *E*^+^, and negative set *E*^−^. In the inference phase, *E*^−^ is empty and the model only needs to rank the entities in *E*^+^ to predict the entity to link. Following this data construction approach, we construct a MIL dataset that contains 500k+ data points from the MedDialog.

### Entity linking model

**Fig. 2:**
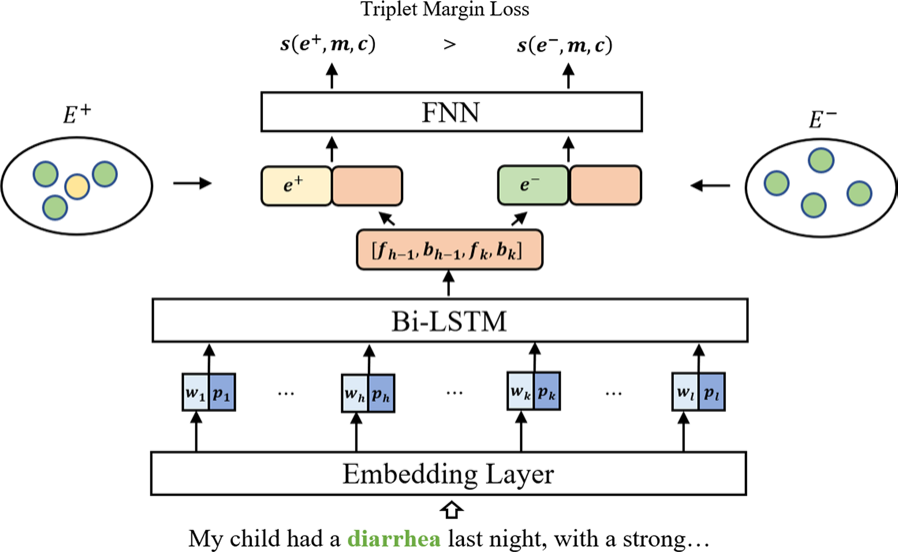
The entity linking model architecture

The entity linking model architecture is shown in Fig. 2.

The model uses a multi-layer BiLSTM [28] as an encoder and an embedding layer to encode the context-mention representation and the ICD10 entity representation respectively. Then the two representation vectors are concatenated, feeding to the following feedforward neural network (FNN) ranking network to score the similarity of the two representation vectors.

Formally, the representation of the context-mention is obtained as follows. For every token $w_i$ in sentence $(w_1,\ldots ,w_l)$, the model gets the token’s word embedding vector $\varvec{w_i}$ and positional embedding vector $\varvec{p_i}$. The word embedding layer is initialized with the pretrained Word2vec weights. Then, the sequence of $[\varvec{w_i},\varvec{p_i}]$ is input into a multi-layer BiLSTM network to encode the sentence. Therefore, the $[\varvec{f_{(h-1)}},\varvec{b_{(h-1)}},\varvec{f_k},\varvec{b_k}]$ from the last layer states output of BiLSTM is used as the context-mention representation, where $\varvec{f_i}$ and $\varvec{b_i}$ are the forward and backward states of BiLSTM.

As for ICD10 entity, we use the average vector of all the tokens’ embedding vectors as its representation. For every token $e_i$ in ICD10 entity string $(e_1,\ldots ,e_l)$, the entity representation $\varvec{e}=avg(E(e_1),\ldots ,E(e_l))$ where *E* is embedding layer initialized with the pretrained Word2vec weights.

After getting the context-mention and the ICD10 entity representation, we concatenate them and feed it into a one-hidden layer FNN to compute score compatibility between a context-mention pair (*m*, *c*) and an entity *e*:

$$\begin{aligned} s(e,m,c)=FNN([\varvec{e},\varvec{f_{(h-1)}},\varvec{b_{(h-1)}},\varvec{f_k},\varvec{b_k}]) \end{aligned}$$

For any context-mention pair (*m*, *c*), the score of correct entity $e^*$ should be higher than the scores of any other entities. Which means, there is at least one candidate entity’s score in *E*^+^ is higher than any candidate entity’s score in the *E*^−^.

### Training

In the training phase, the model learns the ability to correctly rank the candidates. Following the MIL training paradigm, we minimize the triplet max-margin loss to learn the model parameters:

1 $$\begin{aligned} loss = \sum _{(m,c) \in D} \left[ \underset{{e \in E^-}}{max} \; s(e,m,c) + \delta - \underset{{e \in E^+}}{max} \; s(e,m,c) \right] _{+} \end{aligned}$$

where $\delta$ is a margin hyperparameter and $[x]_+=x$ when $x>0$ else $[x]_+=0$; *D* is the constructed MIL data points.

Here, our model relies on a Basic Entity Linking method (BEL) and is designed to enhance the BEL’s performance in a MIL manner. Therefore, before the MIL training procedure, we use the BEL to pretrain the linking model to obtain the basic ranking performance from the BEL. When it comes to MIL training stage, the pretrained model will be trained using the constructed MIL dataset to enhance the BEL’s entity linking ability.

In our method, we need choose a fundamental unsupervised semantic similarity method as our BEL. BEL is used for two causes:

- Pre-train the linking model. During pre-training phase, the top1 entity generated by BEL is treated as the pseudo gold entity, and the model is trained with those mention pseudo gold entity pairs.
- Provide a signal to construct the *E*^+^ and *E*^−^. The methods to construct the *E*^+^ and *E*^−^ need a similarity metric signal which is provided by the BEL.

For simplicity, we use the public pre-trained Word2vec [29] trained on Baidu Encyclopedia corpus [30] as our BEL to calculate the semantic similarity between the symptom mention and the ICD10 entity. For a mention or entity, its representation vector is obtained by averaging the embedding vectors of all tokens in the mention or entity. All ICD10 entities’ vectors are pre-calculated and cached.

## Results

### Preprocessing of MedDialog

MedDialog is an unlabeled dataset crawled from Haodaifu [31] website which is an online healthcare services for medical consultation to doctors. Each consultation in this dataset consists of three parts: (1) description of patient’s medical condition and history; (2) conversation between patient and doctor; (3) (optional) diagnosis and treatment suggestions given by the doctor. Here we use some rules to abstract the second description text field as the sentences to construct the MIL dataset since description text is relatively more concise and informative compared with the conversation text field.

### Experiments setting

Two professional doctors annotate the validation and test datasets. First, we randomly pick a group of sentences from the preprocessed MedDialog dataset and detect the mention spans in the sentences with the same approach as building the MIL dataset, leading to 2000 sentences labeled with mention span. Then the doctors link those 2,000 mentions to their corresponding gold ICD10 entities. Among those mentions, there are 320 mentions which cannot align to any ICD10 entity, 288 mentions that their surface strings match the ICD entity strings exactly. Since we do not deal with the NIL problem and the exact matching cases can be solved with simple string match, we only use the left 1392 annotated mentions and split them into validation and test datasets evenly.

In our experiments, the model is trained with batch size of 100, margin of 0.1, learning rate of $1e^{-3}$ with the Adam optimizer. As to the encoders, for Bi-LSTM, a 2-layer 100 hidden dimension bi-directional LSTM is used.

We use the accuracy as the evaluation for the medical entity linking. That is, the ratio of the correctly linked mention-entity pairs to all mention-entity pairs.

### Experiments results and analysis

The experimental results under different experiment setting are list in Table 2. The *E*^+^ column gives the way to construct the positive candidate set, either *TopK* or *ClusterK*. The *E*^−^ column denotes the negative set constructing way, either *Random All* or *Random TopN*. We use EMEL to refer the Enhanced Medical Entity Linking.

**Table 2:** Accuracy for different experiment setting

	*E*^+^	*E*^−^	Train stage	Accuracy (%)
BEL	–	–	–	58.62
Baseline	Top1	Random Top20	Pre-train	58.62
EMEL-top	Top3	Random Top20	MIL	59.77
	Top3	Random all	MIL	58.76
	Top4	Random Top20	MIL	59.19
	Top4	Random all	MIL	57.90
EMEL-cluster	Cluster2	Random Top20	MIL	59.19
	Cluster3	Random Top20	MIL	**60.34**
	Cluster4	Random Top20	MIL	60.05

Bold value represents the best experimental result

From the experimental results, we can obtain the following observations and analysis.

- Baseline
  We denote the model as a baseline model when the model is pre-trained with the signal from the BEL (basic Word2vec similarity Entity Linking method). In our experiment, the baseline model converges to the same accuracy as the BEL, successfully acquiring the ability of BEL.
- EMEL-top
  EMEL-top means that the model is trained on the MIL data points which *E*^+^ is generated with *TopK* selection method. From the four experiments results in this block, we can observe that *Random TopN* is always better than *Random All* for generating *E*^−^. Therefore, we only use *Random TopN* to generate *E*^−^ in the following EMEL-cluster experiments. Three models get accuracy improved while one model, which uses *Top4* to generate *E*^+^ and *Random All* to generate *E*^−^ witnesses an accuracy decline, dropping from 58.62% to 57.90%. In this declining case, the entity pair from *E*^+^ and *E*^−^ are constructed in a manner that the distance between positive entity and negative entity is so far that the baseline model literally cannot learn any useful information from such soft adversarial pair.
- EMEL-cluster
  EMEL-cluster trains the model on the MIL data points which *E*^+^ is generated with *ClusterK* selection method. By incorporating the ICD10 hierarchy structure in generating *E*^+^ and selecting harder negative entities with *Random Top20*, we can produce a much ambiguous positive-negative entity pair, and drive the model to distinguish the hard cases. The *Cluster3* setting experiment achieved the best enhancing result of 60.34%, further demonstrating the effectivity of leveraging the structural information of ICD10.

### Size of candidate selection pool

**Fig. 3:**
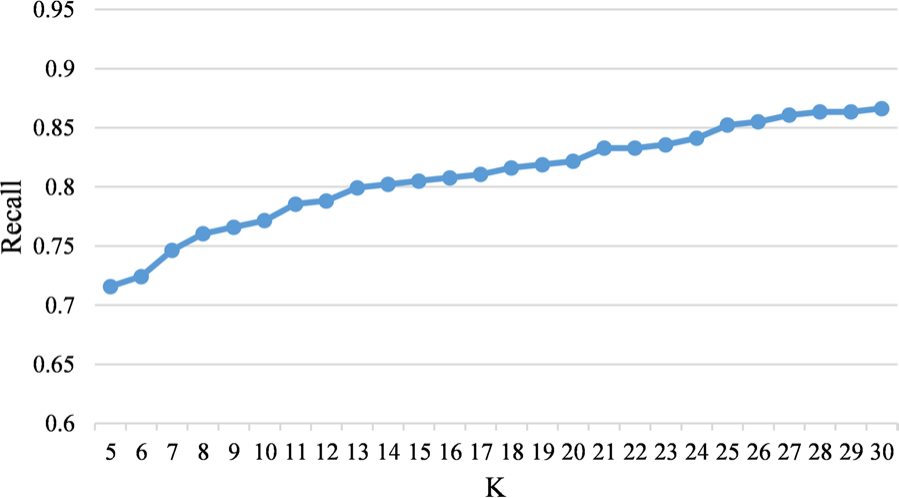
The recall of candidate pool under different K values

Figure 3 shows the recall of the candidate selection pool under different *N* values. As the chart shows, the gold ICD10 entity recall does not change significantly when the *N* comes above 20. Therefore, in our experiments, we use Top 20 similar ICD10 entities from BEL as the selection pool in the candidate generation stage.

## Discussion

The accuracy of 60.34% which our model achieved doesn’t stand out, still remaining a considerable gap between the supervised methods. Derived from the real word application scenario, our method intends to tackle the medical entity linking under the annotation lacking and sparse KB circumstance.

There are three factors hinder the model’s performance:

- Though the size of the symptom dictionary is considerable large, the symptom dictionary only helps in capturing the formal normalized symptom mention, while lots of colloquial unnormalized mentions like “did not fall asleep” (insomnia formally) are missed. Such omission of colloquial mentions leads to a homogeneous mention distribution MIL dataset.
- The BEL brings too many noises in constructing the *E*^+^ and *E*^−^. In our experiments, the recall of the candidate pool reaches around 80%, which means nearly one-fifth data points in the MIL training data is noisy. Those data points’ *E*^+^ just fail to include the gold entity. The model can be confused by the conflict criteria between noisy data points and correct data points.
- The insufficient representation of the ICD10 entity also undermines the model’s performance. An expressive, dense and robust representation for the context-mention and entity is the key point to keep the model to learn a stable ability to distinguish between the positive and negative entity. In contrast with context-mention, the representation learning of the ICD10 entity is much harder. The shortage of information in ICD10 entity surface string and intrinsic relation sparsity in the ICD10 classification schema hinder the attempts to get an expressive entity representation. We list some error cases in Table 3. As the error cases show, the top 5 candidate entities are very similar in their Chinese surface strings. The most difficult cases are generally the adjacent entities in the same cluster with the gold entity, i.e. the hypernym entities or hyponym entities.

**Table 3:**
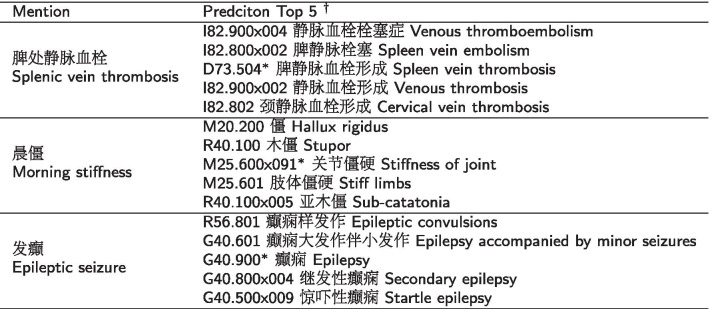
Error analysis examples

$^{\dagger }$ We use a more fine-grained localized Chinese version of ICD10: The Disease Classification and Code National Clinical Edition v2.0. Therefore there may exit one more classification after the sub-classification, such as x002 in the Table 3. Correct gold candidate entities are indicated with an asterisk$^*$.

Those shortcomings can be alleviated with further improvements. Using a colloquial medical mentions NER tool will help to the MIL dataset construction. A stronger BEL and the co-occurrence statistics between mention and entity can be used to reduce the noise in candidate generation. Last, pre-trained language models like Bert [32] and ELMo [33] trained on large specific domain datasets like PubMeb [34] and MIMIC III [35] also can be used to get a stronger representation for the mention-context and ICD10 entity [36, 37].

## Conclusions

We propose an unsupervised entity linking method to the entity linking in the medical domain, using MIL training manner. The experimental results show that our method indeed enhances the fundamental BEL’s performance.

## Data Availability

The MedDialog dataset is open and free accessed. The labeled dataset are not publicly available due to the company’s regulations, but are available from the first author on reasonable request.

## Abbreviations

EL: Entity linking
MIL: Multi-instance learning
KB: Knowledge base
FNN: Feedforward neural networks
BiLSTM: Bidirectional long short-term memory
ICD: International classification of diseases
MeSH: Medical subject headings
UMLS: Unified medical language system
SNOMED CT: Systematized nomenclature of medicine - clinical terms
